# Weight Status Influences Carotid Intima-Media Thickness to Executive Function Among Older Adults

**DOI:** 10.1093/geroni/igaa057.1183

**Published:** 2020-12-16

**Authors:** Desiree Bygrave, Regina Wright

**Affiliations:** University of Delaware, Newark, Delaware, United States

## Abstract

Carotid atherosclerosis has emerged as an early predictor of reduced cognitive function. Underlying this association are risk factors, such as overweight and obesity, that promote carotid atherosclerosis and poor cognitive outcomes. Given the prevalence of overweight and obesity among older adults, there is a critical need to better understand how atherosclerosis influences cognitive function in the context of elevated weight. To address this gap, the current study examined relations between carotid atherosclerosis (carotid intima-media thickness [IMT]), and attention (Trailmaking Test) and executive function (Verbal Fluency Test) performance, and whether they varied as a function of weight status (body mass index [BMI] classification). Data were analyzed from 162 older adults (mean age = 68.43y, 34% male, 41% African American), free of major disease. Mutliple regression and analysis of variance analyses, adjusted for age, sex, education and mean arterial pressure, showed a statistically significant IMT x BMI interaction for Verbal Fluency performance (p=.04) and a trending IMT x BMI interaction for Trailmaking A performance (p=.05). Simple effects analysis of IMT and Verbal Fluency performance showed that this association was most pronounced among those who are obese. Findings suggest atherosclerosis may influence executive function in the context of obesity among older adults. As the development of carotid atherosclerosis is strongly related to aging, our findings suggest that maintaining a healthy weight may reduce its impact on executive function in older adulthood.

